# Curcumin Mitigates Accelerated Aging after Irradiation in *Drosophila* by Reducing Oxidative Stress

**DOI:** 10.1155/2015/425380

**Published:** 2015-03-01

**Authors:** Ki Moon Seong, Mira Yu, Kyu-Sun Lee, Sunhoo Park, Young Woo Jin, Kyung-Jin Min

**Affiliations:** ^1^National Radiation Emergency Medical Center, Korea Institute of Radiological & Medical Sciences, Seoul 139-706, Republic of Korea; ^2^Department of Biological Sciences, Inha University, 100 Inha Street, Incheon 402-751, Republic of Korea; ^3^Bionanotechnology Research Center, Korean Research Institute of Bioscience and Biotechnology, Daejeon 305-806, Republic of Korea

## Abstract

Curcumin, belonging to a class of natural phenol compounds, has been extensively studied due to its antioxidative, anticancer, anti-inflammatory, and antineurodegenerative effects. Recently, it has been shown to exert dual activities after irradiation, radioprotection, and radiosensitization. Here, we investigated the protective effect of curcumin against radiation damage using *D. melanogaster*. Pretreatment with curcumin (100 *μ*M) recovered the shortened lifespan caused by irradiation and increased eclosion rate. Flies subjected to high-dose irradiation showed a mutant phenotype of outstretched wings, whereas curcumin pretreatment reduced incidence of the mutant phenotype. Protein carbonylation and formation of *γ*H2Ax foci both increased following high-dose irradiation most likely due to generation of reactive oxygen species. Curcumin pretreatment reduced the amount of protein carbonylation as well as formation of *γ*H2Ax foci. Therefore, we suggest that curcumin acts as an oxidative stress reducer as well as an effective protective agent against radiation damage.

## 1. Introduction

Out of several hundred aging theories, the most popular aging theory is oxidative stress theory of aging. It claims that aging is caused by oxidative damage to macromolecules. Oxidative stress is the result of an imbalance between generation of reactive oxygen species by essential life systems and detoxification of reactive radicals by defense mechanisms within organisms [1]. Disruption of the normal redox state of cells induces cytotoxic effects through production of reactive intermediates, which inflict damage to all cellular components, including proteins, lipids, and DNA [2]. Reactive oxygen species such as superoxide (O_2_^•−^), hydroxyl (OH^•^), peroxyl (RO_2_^•^), and hydroperoxyl (HO_2_^•^) are generated by natural respiration in animals and environmental stresses such as radiation, chemicals, and heat [3, 4].

Although the biological effects of low-dose radiation less than 100 mSv have not been fully established, exposure to high-dose radiation caused by unexpected accidents related to artificial sources has many deleterious consequences in humans, including organ malfunction, malignant cancer development, genetic mutagenesis, and developmental abnormalities [5–7]. Moreover, ionizing radiation has long been used as a standard medical treatment to kill cancer cells and shrink tumors [8]. Cancer radiotherapy destroys chromosomes by making it impossible for them to proliferate. Normal cells are also damaged by this therapy, which is the main drawback of this medical procedure. Several antioxidative natural extracts have been combined together in order to reduce radiation injury and protect normal cells [9]. For example, melatonin has been shown to imbue significant radiation protection against chromosomal aberrations and micronuclei formation when administered to mice prior to radiation exposure [10]. Further, several flavonoid compounds such as quercetin, myricetin, and orientin have been reported as potent antioxidants with radioprotective ability [11]. Resveratrol, a polyphenolic plant product, was also shown to attenuate radiation damage in* C. elegans* by scavenging ROS [12].

Curcumin derived from turmeric is a representative plant phenolic compound possessing therapeutic properties [13, 14]. It is known to eliminate oxygen free radicals, inhibit lipid peroxidation, and protect cellular macromolecules such as DNA from oxidative stress [15, 16]. Curcumin has been shown to reduce chromosomal aberrations in models of human breast cancer, probably due to its antioxidative activity [17]. Fruit flies fed a curcumin diet have shown an extended lifespan, improved health, and modulated expression of aging-associated genes [18, 19].

Due to its antioxidative activity, curcumin has been proposed as a radiation protector. Pretreatment with curcumin has been shown to protect lymphocytes against *γ*-radiation-induced cellular damage [15]. Curcumin also was found to protect against cutaneous radiation-induced damage in mice [20]. However, several previous studies showed that curcumin has no protective effect against the clastogenicity of *γ*-radiation [21–23]. Therefore, it remains unclear whether or not curcumin indeed acts as a radiation protector. Moreover, most studies on the radioprotective effects of curcumin have been performed at the cellular level [24–26]. Studies using model animals would more strongly support the conclusion that curcumin protects against radiation damage. Therefore, we evaluated the protective effect of curcumin against ionizing radiation using* D. melanogaster* and found that curcumin may be effective as a radiation protector.

## 2. Materials and Methods

### 2.1. Fly Husbandry

We performed all experiments using wild-type Canton-S flies. Larvae of the Canton-S strain were grown on standard cornmeal-sugar-yeast (CSY) medium (5.2 g of cornmeal, 11 g of sucrose, 11 g of yeast [MP Biomedicals, Solon, OH], 1.1 mL of 20% tegosept, and 0.79 g of agar per 100 mL of water) supplemented with several grains of live yeast. The rearing room was maintained at 25°C with 45% humidity on a 12 h : 12 h light-dark cycle.

### 2.2. Curcumin Pretreatment

Stock solution of curcumin (5 mM) was prepared dissolving curcumin (218580100, Acros Organics) in 99% ethanol and was supplemented to sucrose-yeast (SY) food at a concentration of 100 *μ*M. Same amount of ethanol was supplemented to food without curcumin. Collected eggs were reared in the SY food containing curcumin before irradiation at the 3rd instar larvae stage.

### 2.3. *γ*-Irradiation Exposure

Eggs were collected from young female flies over 12–14 h and reared on SY medium. 3rd instar larvae were irradiated in a *γ*-irradiation machine (^137^Cs, IBL 437N; CIS Bio International, Gifsur-Yvette, France) at a dose rate of 0.8 Gy/min. Following irradiation, nonirradiated and irradiated flies were maintained contemporaneously in the same incubator at 25°C.

### 2.4. Pupation and Eclosion Rates

Irradiated larvae were checked daily to determine pupation and eclosion rates. Pupation rate was calculated based on the total number of pupae divided by the number of larvae, whereas eclosion frequency was calculated based on the total number of eclosed flies divided by the number of larvae.

### 2.5. Lifespan

When irradiated larvae were eclosed, adult flies were collected over 48 h and randomly assigned to 500 mL demography cages to achieve a final density of 100 females and 100 males per cage. SY diets were prepared with 10 g of sucrose, 10 g of yeast, 1.1 mL of 20% tegosept (w/v in ethanol), and 0.79 g of agar per 100 mL of water. The vials containing SY diets were changed every 2 days, and all mortalities were recorded. Three replicates were established for each dose level.

### 2.6. Detection of Protein Oxidation (Protein Carbonylation)

Protein carbonylation was measured using an OxyBlot protein oxidation detection kit according to the manufacturer's instructions (Millipore). Briefly, radiation-exposed larvae under each condition were homogenized in lysis buffer (50 mM Tris-HCL pH 7.4, 150 mM NaCl with protease inhibitor cocktail). For the positive control, protein sample was prepared from larvae fed 20 mM paraquat for 16 h. Protein samples were then treated with 2,4-dinitrophenylhydrazine (DNPH). Reaction of DNPH with carbonylated proteins allows the formation of 2-4-dinitrophenylhydrazone (DNP), which can be detected with anti-DNP antibody. Samples were subjected to 10% SDS-PAGE and transferred onto a PVDF membrane (Roche). DNP groups were then immunodetected with rabbit anti-DNP antibody, followed by secondary anti-HRP antibody and ECL revelation. To normalize protein loading, the transferred SDS-PAGE gel was stained with Coomassie blue.

### 2.7. *γ*H2Ax Foci Staining

To detect double-strand breaks, irradiated larvae were dissected in cold PBS and fixed for 20 min at room temperature in PBS containing 4% paraformaldehyde. After washing and blocking with PBS containing 0.1% Triton and 2% BSA, wing imaginal discs were incubated with antiphosphorylated H2Ax (*γ*H2Ax, Upstate Biotechnology). For visualization, samples were mounted in VECTASHIELD Mounting Media (Vector Lab), and fluorescence images were acquired using a FluoView confocal microscope (Olympus).

### 2.8. Statistical Analyses

All demographic data were presented as the mean ± SEM and analyzed with a one-way analysis of variance (ANOVA) on ranked data using standard survival models in the JMP statistical package and Prism software (GraphPad, La Jolla, CA). Asterisk indicates significant difference from the control (^**^ *P* < 0.001 and ^*^ *P* < 0.05). The tests used and sample sizes for each experiment are indicated.

## 3. Results 

### 3.1. Effect of Curcumin Pretreatment on* Drosophila* Lifespan after Radiation Exposure

**Figure 1:**
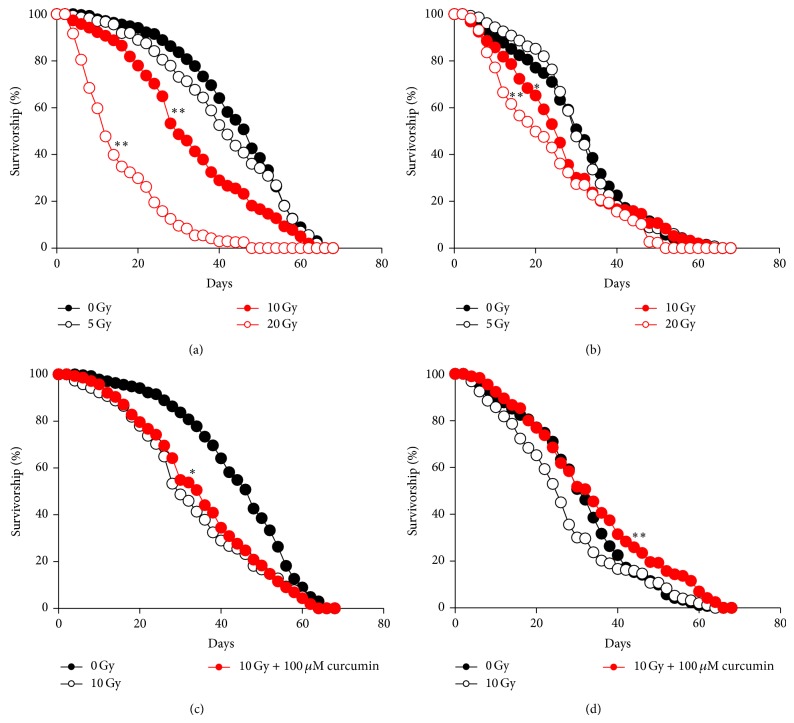
Curcumin pretreatment recovers shortened fly lifespan by ionizing radiation. Several doses of ionizing radiation were administered at the 3rd larval stage, and the lifespans of adult males (a) and females (b) were measured. Larvae were fed 100 *μ*M curcumin from egg hatching before 10 Gy of irradiation at the 3rd larval stage, and the lifespans of adult males (c) and females (d) were measured (^*^ *P* < 0.05, ^**^ *P* < 0.01).

Previous studies have reported that ionizing radiation reduces the lifespan of* Drosophila *to various degrees depending on the irradiation dosage and strain genetic background [27, 28]. Here, we first subjected larvae of fruit flies to irradiation at several doses and then recorded lifespans of adults in order to determine the optimal dose to analyze the effects of curcumin (Figures 1(a) and 1(b)). The effect of curcumin pretreatment was evaluated in flies irradiated at 10 Gy, which showed a mean lifespan of approximately 30 days in both males and females (Table 1). We reared Canton-S flies after egg hatching with fly medium containing 100 *μ*M curcumin, and ionizing radiation was administered at the 3rd instar larva stage. 100 *μ*M curcumin was chosen as the most effective dose based on preliminary experiment. Flies pretreated with curcumin showed significant extension of their mean lifespan—5.5% for males (*P* < 0.01) and 26.5% for females (*P* < 0.01) (Table 1, Figures 1(c), 1(d)). These data indicate that curcumin pretreatment extended the lifespan of irradiated flies by mitigating the harmful effects of ionizing radiation.

### 3.2. Effect of Curcumin Pretreatment on* Drosophila* Development after Radiation Exposure

**Figure 2:**
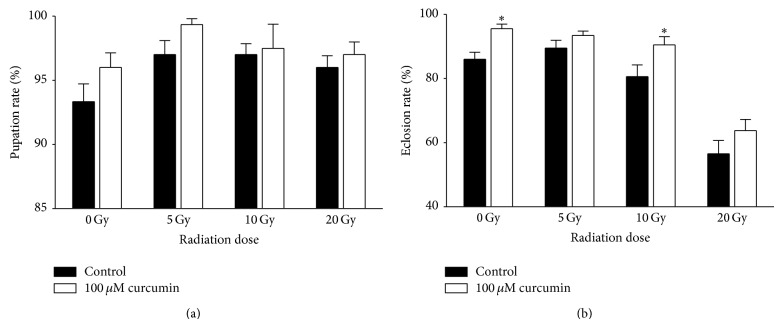
Curcumin pretreatment increases the eclosion rate of fruit flies. Pupation rate (a) and eclosion rate (b) of irradiated flies were recorded as described in the materials and methods section. Pretreatment with curcumin improved the eclosion rate in some treatments (^*^ *P* < 0.05).

All insects, including* Drosophila*, undergo marked morphological changes during their development to adult stage known as metamorphosis, which is an excellent parameter to detect physiological effects following environmental fluctuation. Here, we measured pupation and eclosion rates of flies pretreated with curcumin after irradiation. The pupation rate of curcumin-pretreated flies was not significantly different after irradiation (*P* > 0.07) (Figure 2(a)). However, the eclosion rate of flies was reduced as the radiation dose increased. The eclosion rate of control flies was 83%, whereas that of flies irradiated at 20 Gy was reduced to 58%. Curcumin pretreatment distinctly augmented the eclosion rate in irradiated flies with statistical significance (*P* < 0.05) (Figure 2(b)). Curcumin pretreatment without irradiation also increased the eclosion rate (*P* < 0.05).

### 3.3. Effect of Curcumin Pretreatment on* Drosophila* Phenotype after Radiation Exposure

**Figure 3:**
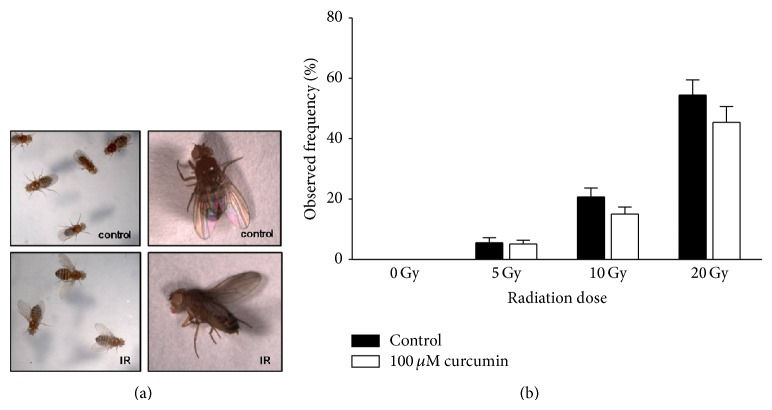
Irradiation increases incidence of flies with outstretched wings. Some irradiated flies emerged with outstretched wings (a). The incidence of flies with outstretched wings increased as the radiation dose increased. Curcumin pretreatment tended to reduce incidence, but the difference was not significant in all treatments.

High-dose irradiation has been shown to induce chromosomal mutations and malformation of external organs [29–31]. Here, we analyzed the specific phonotype caused by irradiation to determine whether or not curcumin reduces the mutagenic effects of ionizing radiation. Irradiation with 20 Gy at the 3rd instar larval stage resulted in outstretched wings on bodies of adult flies (Figure 3(a)), and the frequency of the mutant phenotype increased as the radiation dose increased (Figure 3(b)). Specifically, no mutant phenotype was observed at 0 Gy of irradiation, whereas about 60% of flies showed the mutant phenotype at 20 Gy of irradiation. Although curcumin pretreatment did not significantly reduce the frequency of mutation, lower frequency of the mutant phenotype was a tendency in all curcumin-pretreated groups (Figure 3(b)).

### 3.4. Effect of Curcumin Pretreatment on ROS Generation after Radiation Exposure

**Figure 4:**
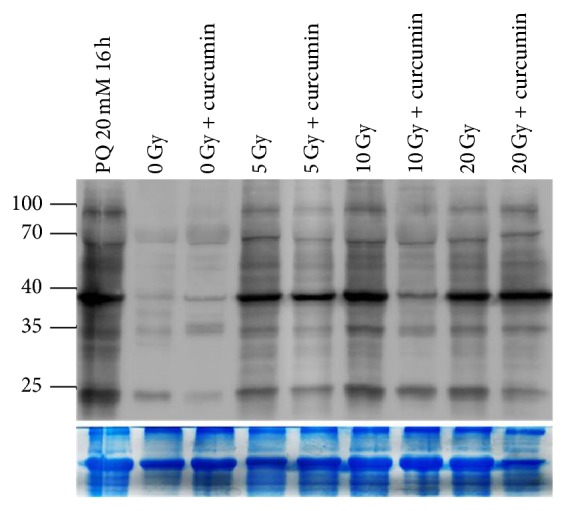
Curcumin pretreatment reduces radiation-induced protein carbonylation. Both paraquat (positive control) and irradiation increased protein carbonylation, whereas curcumin pretreatment decreased protein carbonylation. A gel image stained with Coomassie blue was used as an internal control of protein loading amount in SDS-PAGE.

The phenotypic data acquired in this study indicate that curcumin reduced the various stresses caused by ionizing radiation. Since it is well known that radiation induces oxidative stress and curcumin is an excellent antioxidant, we examined whether or not curcumin detoxifies radiation-induced oxidative damage. Protein carbonylation is known to be a key biomarker of oxidative stress generated by carbonyl (CO) groups (aldehydes and ketones), which are produced on protein side chains, especially in proline, arginine, lysine, and threonine, following their oxidation [32]. Here, we extracted protein lysates from flies and performed protein carbonylation assay as described in Section 2. Protein carbonylation increased upon irradiation, whereas curcumin pretreatment obviously reversed this in irradiated flies. Paraquat, known to be a chemical inducing cellular protein carbonylation, was used as a positive control (Figure 4).

### 3.5. Effect of Curcumin Pretreatment on DNA Damage after Radiation Exposure

**Figure 5:**
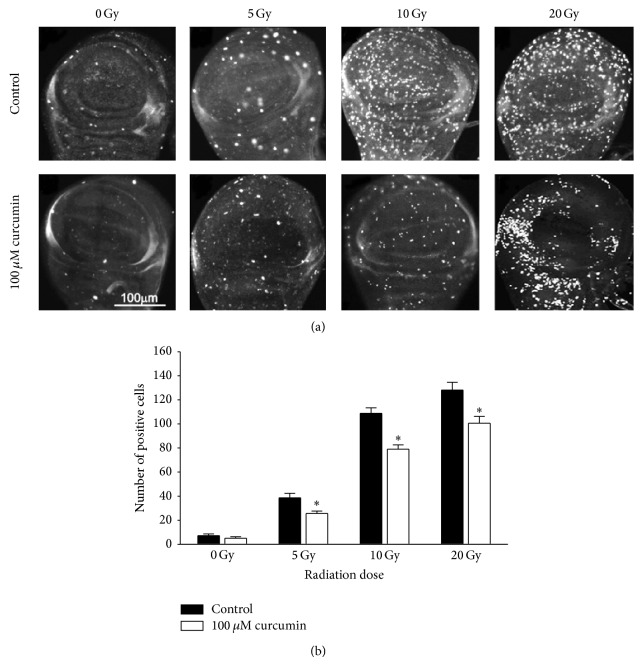
Curcumin reduces formation of radiation-induced *γ*H2Ax foci. Phosphorylated H2Ax was used as a marker of DNA double-strand breaks. Foci on wing discs were detected by immunostaining with specific antibodies for *γ*H2Ax (a). The incidence was measured by counting spots (b) and analyzed statistically (^*^ *P* < 0.05).

Reduced oxidative stress by curcumin could diminish the damage inflicted by ionizing radiation. DNA double-strand breaks caused by radiation-induced ROS impair normal cellular survival. In mammal, phosphorylated H2Ax (*γ*H2Ax) foci, an indicator of DNA double-strand breaks, are found in the nucleosomes near radiolytic damaged region [33]. Since antibody to mammalian *γ*H2Ax can recognize* Drosophila γ*H2Av based on sequence homology [34], we monitored the radiation-mediated DNA damage with *γ*H2Ax foci in larval wing disc. Here, ionizing radiation induced DNA breaks in a dose-dependent manner, whereas curcumin pretreatment significantly reduced formation of *γ*H2Ax foci in the larval wing disc of* Drosophila* (Figure 5). These data indicate that curcumin reduced radiation induced the genome instability in* Drosophila* by increasing resistance to oxidative stress.

## 4. Discussion

In this paper, we presented data showing that curcumin reversed the shortened lifespan of irradiated flies as well as increased the eclosion rate. Curcumin also attenuated oxidative stress and DNA alterations caused by ionizing radiation. Interestingly, irradiation caused a larger reduction in lifespan in males than in females, whereas curcumin pretreatment was more effective in females than in males (Figure 1). This sexually dimorphic difference may be due to differential hormonal regulation of male and female fecundity [35–37]. It may also be due to gender differences in susceptibility to oxidative stress between males and females. Some parameters of free radical processes are different between male and female* Drosophila*. For example, a previous study showed differences in oxygen consumption of extracted mitochondria and mitochondrial DNA copy number between male and female* Drosophila* [37].

To our knowledge, this is the first report showing that high-dose irradiation of larvae results in an abnormal outstretched wing phenotype (Figure 3). Generally,* Drosophila* adults are quite resistant to irradiation. Even 500 Gy of radiation has been shown to have little effect on adult survival following irradiation at the adult stage (unpublished data), which may be due to the cuticular exoskeleton of flies. Unlike adults,* Drosophila* larvae are susceptible to irradiation due to their soft cuticular structure. As mentioned above, in this study, lifespans were greatly reduced and incidence of the outstretched wing phenotype increased as the radiation dose increased. Actually, half of the flies emerged with the outstretched wing phenotype when 20 Gy of radiation was administered to 3rd instar larvae. It remains unknown which signaling pathway is involved in the formation of the outstretched wing phenotype, but we suspect the JAK/STAT signaling pathway since it participates in the formation of the imaginal wing disc [38, 39]. Further investigation is necessary to determine the molecular mechanisms of outstretched wing formation after irradiation.

Eclosion rates of nonirradiated or irradiated flies were improved by curcumin pretreatment (Figure 2). As an explanation, curcumin has the potential to remove ROS generated during development and/or radiation exposure. A previous report of delayed aging upon curcumin treatment supports our observations since aging is tightly coupled with ROS generation [18]. Both 20 mM paraquat and irradiation increased protein carbonylation, which itself was reduced by curcumin treatment (Figure 4). Similarly, pretreatment with curcumin to irradiated lymphocytes reduced lipid peroxidation and increased antioxidative properties, thereby preventing injury to lymphocytes [15]. Overall, curcumin provided* Drosophila* with augmented resistance to overcome radiation-induced oxidative stresses.

Collectively, these effects of curcumin may be due to its scavenging activity and distinct structural characteristics. First, curcumin has a hydrophobic structure that allows it to easily pass through the plasma membrane into the cytoplasm, where it can scavenge ROS more easily than hydrophobic molecules [40]. Second, curcumin has electron-donating groups such as phenolic hydroxyl groups and a *β*-diketone structure responsible for removing free radicals from cells [15]. Increased resistance to oxidative stress by curcumin could be attributed to its transcriptional regulation; namely, curcumin can activate transcriptional factors and increase the expression of genes involved in oxidative defense [41, 42].

However, some scientists have remarked that curcumin could be a “double-edged sword,” similar to other herbal antioxidants in tumorigenesis [43, 44]. The carcinogenic or prooxidant effects of curcumin have been shown to be mediated by mechanisms such as iron depletion, inhibition of cytochrome p450, and interference with the p53 tumor suppressor pathway [40, 45, 46]. Moreover, other lines of evidence raise concerns about the safety of curcumin for cancer treatment. Specifically, curcumin shows diverse clinical effects depending on its concentration [47]. To develop curcumin into a preventive or therapeutic drug, the optimal dose that elicits only desirable effects should be determined.

The development of radioprotectors is an area of great significance due to its wide applications in planned radiotherapy as well as unexpected radiation exposure. Although some conflicting behaviors of curcumin on radioprotective function were reported, there are number of studies showing that curcumin offers protection to normal cells from radiation [15, 20, 48]. Our present data using* Drosophila* prove that curcumin improved radioresistance by relieving oxidative stress, thereby consolidating the radioprotective effects of curcumin.

## 5. Conclusions

In this paper, we have presented data showing that that curcumin relieves the oxidative stress and DNA damage caused by high-dose radiation in* Drosophila*. Curcumin pretreatment extends lifespan and decreases the frequency of mutagenic phenotype caused by ionizing radiation. Given antiaging benefits of curcumin from antioxidative properties, it will be of interest to determine whether curcumin can be used as a radioprotective agent in mammalian models.

## Figures and Tables

**Table 1:** Mean longevity recovery by pretreatment of curcumin.

Treatment	Flies numbers	Mean longevity	SD	*P* value
Male				
Control	270	44.6889	0.82040	—
IR^a^	259	33.2819	0.96298	<0.0001^*^
IR + curcumin^b^	279	35.1183	0.89536	<0.0001^**^
Female				
Control	262	31.1908	0.84744	—
IR^a^	253	27.0119	0.93228	0.0151^*^
IR + curcumin^b^	286	34.1748	0.95931	<0.0001^**^

^a^Radiation at 10 Gy was irradiated at 3rd instar larval stage.

^b^Flies were cultivated in the medium containing 100 *μ*M of curcumin until irradiation.

^*^Compared with control, ^**^compared with IR group.
